# Grouping Mechanisms in Numerosity Perception

**DOI:** 10.1162/opmi_a_00037

**Published:** 2020-11-01

**Authors:** Lorenzo Ciccione, Stanislas Dehaene

**Affiliations:** Cognitive Neuroimaging Unit, CEA DSV/I2BM, INSERM, Université Paris Sud, Université Paris-Saclay, NeuroSpin Center, Gif-sur-Yvette, France; Collège de France, Paris, France; PSL University, Paris, France; Cognitive Neuroimaging Unit, CEA DSV/I2BM, INSERM, Université Paris Sud, Université Paris-Saclay, NeuroSpin Center, Gif-sur-Yvette, France; Collège de France, Paris, France

**Keywords:** numerical cognition, arithmetics, mathematics, groupitizing, multiplication

## Abstract

Enumeration of a dot array is faster and easier if the items form recognizable subgroups. This phenomenon, which has been termed “groupitizing,” appears in children after one year of formal education and correlates with arithmetic abilities. We formulated and tested the hypothesis that groupitizing reflects an ability to sidestep counting by using arithmetic shortcuts, for instance, using the grouping structure to add or multiply rather than just count. Three groups of students with different levels of familiarity with mathematics were asked to name the numerosity of sets of 1–15 dots in various arrangements, for instance, 9 represented as a single group of 9 items, three distinct groups of 2, 3, and 4 items (affording addition 2 + 3 + 4), or three identical groups of 3 items (affording multiplication 3 × 3). Grouping systematically improved enumeration performance, regardless of whether the items were grouped spatially or by color alone, but only when an array was divided into subgroups with the same number of items. Response times and error patterns supported the hypothesis of a multiplication process. Our results demonstrate that even a simple enumeration task involves mental arithmetic.

## INTRODUCTION

Understanding the cognitive basis of numerosity perception is a central topic in the field of numerical cognition. A broad divide separates approximate versus exact numerosity perception. Approximating the cardinal of a set of objects is an ancient and evolutionarily useful process, common to many animal species (Jordan et al., 2008; McComb et al., 1994; Rugani et al., 2015) and to all human cultures, independent of formal education (Gordon, 2004; Pica et al., 2004). Finding the exact numerosity of a large set, however, is a distinct ability, which seems only present in those human cultures that possess a set of counting symbols that allows them to assign, with a 1:1 correspondence, a specific name to each specific cardinal value of a set (Dehaene et al., 1999; Gelman & Gallistel, 1978; Pica et al., 2004). Determining the exact numerosity of a large set requires a counting strategy, that is, the pairing of objects with the series of number symbols in an incremental 1:1 manner. Counting is evidenced by a systematic, linear increase in naming times as a function of numerosity, suggesting a serial process (Mandler & Shebo, 1982).

Beyond counting, humans also possess another mechanism of exact numerosity assessment, subitizing. It was long observed that, for small groups of one, two, or three elements, human adults do not need a counting strategy to determine their cardinal value, but they can embrace it at once (Jevons, 1871), as if our sensory system were able to determine the “twoness” or the “threeness” of a set without considering each item separately. This ability was first scientifically analyzed by Kaufman and collaborators in 1949 (Kaufmann et al., 1949), who called it “subitizing,” from the medieval Latin *subitare*, which means understanding something immediately, without reflection. The term is thus used to indicate the rapid, confident, and accurate numerosity judgments of sets composed of three items or less. Later on, it was shown that this limit could be overcome via repeated practice with fixed patterns (Wolters et al., 1987) or by using canonical patterns such as dice patterns instead of random configurations (Mandler & Shebo, 1982), suggesting that “adults first develop simple canonical perceptions for twoness and threeness and then apply these schemas to the counting of large arrays.”

While counting and subitizing are considered the two main processes underlying human exact enumeration, Wender and Rothkegel (2000) and Starkey and McCandliss (2014) studied a third process: grouping. They found that the classical set size effect observed for numerosities above three (a strong increase of enumeration latencies with numerosity) essentially vanishes when the items can be grouped into smaller subsets. The grouping cue that they examined consisted in the spatial separation of dots into distinct subgroups, each with a numerosity in the subitizable range of one to three items. The ability to capitalize on grouping information in order to facilitate the enumeration process was termed “groupitizing.” Starkey and McCandliss (2014) further proposed that groupitizing might “reflect adults’ ability to use their grasp of number concepts such as the knowledge that specific numbers are composed of specific subsets.” In support of this conclusion, they showed that groupitizing was not present in a younger group of kindergartners, that the size of the effect increased with age, and that its amplitude correlated with arithmetic abilities in classical symbolic arithmetic tasks. However, they did not analyze the nature of the groupitizing process itself.

The goal of the present research is to fill this gap by providing a thorough exploration of the conditions under which groupitizing occurs in adult subjects, and to explore its relation to symbolic arithmetic. In their studies, Wender and Rothkegel (2000) and Starkey and McCandliss (2014) only created groups of subitizable items by spacing them apart. However, is spatial distance the only cue that can induce groupitizing? Furthermore, what is the role (if any) of the recognition of repeated patterns within the array? If arithmetic is involved, then we should predict faster naming times when the grouping supports mental multiplication, because the items are grouped in groups of equal sizes (e.g., 6 items = 3 groups of 2 items = 3 × 2).

More specifically, we asked the following four questions:

1)  Do repeated groupings with the same number of items facilitate groupitizing? The mental multiplication hypothesis predicts that arrays divided into equal subsets (e.g., nine dots divided into three groups of three) should be faster enumerated than arrays divided into nonequal subsets (e.g., nine dots divided into groups of four, three and two dots), because the former display facilitates a multiplication process. Furthermore, this effect should be maximal when the subgroups share not only the same numerosity, but also the same shape, such as that it is more immediately obvious that they share the same numerosity and that the total number can be immediately obtained by multiplying by the number of groups.
2)  Can color and spatial contiguity act as groupitizing cues? Since both color and distance are well-known cues that promote grouping, in agreement with Gestalt theories of perception (Brunswik & Kamiya, 1953; Wagemans et al., 2012), if groupitizing reflects an abstract arithmetic process, it should be deployed identically whether the items can be grouped spatially or by color (e.g., six items = two red, two blue, and two green). Furthermore, past their distinct perceptual stage, spatial and color groupings should show additive effects of other grouping variables.
3)  Which cognitive computations underlie groupitizing? If multiplication and addition are involved, depending on the specific array patterns, we predict different patterns of response times (RT) and error rates for displays that afford (1) addition only, for example, 6 = 1 + 2 + 3; (2) multiplication, for example, 6 = 2 + 2 + 2 = 2 × 3; or (3) a combination of both, for example, 7 = 3 + 3 + 1 = 2 × 3 + 1. Specifically, we predict that multiplication should afford considerable savings in effort, response time, and error rate. We also predict that this could occur at the expense of the emergence of a new error type: for displays affording multiplication, we should observe table errors (Campbell & Graham, 1985), that is, a slip to the next line or column of the multiplication table (e.g., 4 × 2 = 6) because subjects make an erroring in the selection of one of the operands or in the retrieval of the correct result from memory. On the other hand, for arrays not affording multiplication, we predict enumeration errors, which typically cluster around the correct numerosity (thus reflecting an error in counting or in approximating the correct numerosity).
4)  Does groupitizing vary with mathematical knowledge? Again, a reliance on arithmetic facts would predict that, for equal age, the participants’ level of math training should affect groupitizing performance.

## METHODS

### Participants

The experiment involved 42 participants with normal (or corrected to normal) vision and no color blindness. We replicated the experiment in three groups of participants with low, medium, or high levels of math knowledge (for a similar approach, see Dehaene et al., 1993). At the highest level, we tested 15 students in mathematics or related fields (physics, chemistry, and informatics) at the highly selective Ecole Normale Supérieure (ENS Ulm, Paris). For the medium level, we tested 15 students in humanities, also at ENS, who never took university-level exams in mathematics or related disciplines. These groups differed in their knowledge of university-level mathematics but they both had excellent performances in basic mathematics (they all received extremely high grades in their high school final mathematics exam [French baccalauréat]: 19.25/20 ± 0.7 for science students and 18.5/20 ± 1.1 for humanities students). As the lowest level, a third group of 12 students were selected among first-year students of the Psychology Department of the Université de Saint Denis (Paris). Note that, in France, entrance to university is a mandatory right and is therefore unselective. The third group had a much smaller familiarity with mathematics and considerably worst performance in their high school final mathematics exam (9.25/20 ± 1.9). It is important to note, as a limitation of our subjects’ selection, that the mathematical knowledge of our sample might correlate with other general cognitive skills (language, problem solving, working memory) that were not assessed here. More work would be needed to precisely disentangle those competences from mathematical knowledge.

The experimental procedure was approved by the local ethical committee, and all subjects gave written consent and were informed that they could withdraw from the experiment at any moment without giving any reason. They were compensated with 12 euros for their 60-min participation in the experiment. All data were treated anonymously. Six subjects out of 30 (three from the humanities group and three from the science group) were excluded from the data analyzes described in the following paragraphs, due to these reasons: two participants were color blind but they did not inform us about it before the experiment; the computer crashed during two other experimental sessions and no data were recorded from these subjects; two other participants failed to perform the task in the correct way, since they answered for more than 50% of the trials after the presentation of the stimulus (during the fixation cross). We thus analyzed data from 36 subjects (12 humanities students, 12 sciences students, and 12 psychology students; age: 21 ± 1.5; 20 females, 16 males).

### Stimuli

Subjects were seated in front of a monitor, with their eyes at a distance of 60 centimeters from the screen. Stimuli were black and colored dots of 3 millimeters diameter (0.29° of visual angle) on a white background; the arrays spanned an area of 12 centimeters squared (11.42° of visual angle), at the center of the screen (similar to Mandler & Shebo, 1982; Starkey & McCandliss, 2014). Arrays comprised between 1 and 15 dots. However, arrays of 1, 2, 3, 13, 14, and 15 dots were presented only as fillers, in order to avoid a distinct pattern of improved performance at the extremes of the range of numbers tested, a phenomenon described by Burr and colleagues (Burr et al., 2010).

**Figure 1.**
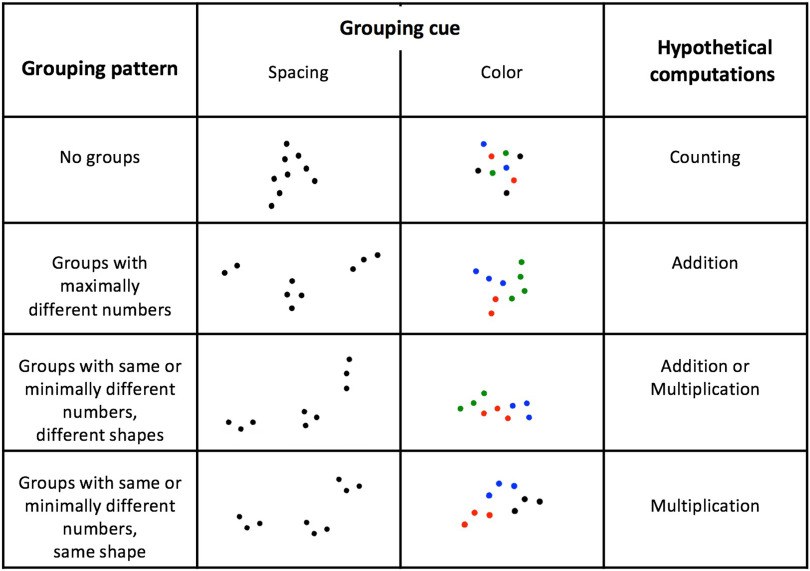
**Stimuli used in the present enumeration task.** The figure shows examples of stimuli for a target numerosity of nine dots.

The design was a 2×4 factorial design where stimuli varied according to the “grouping cue” factor (two levels: spacing and color) and the “grouping pattern” factor (four levels; see Figure 1). In the “spacing” grouping-cue condition, arrays comprised between two and four spatially separated subgroups, each with one, two, three, or four dots. The minimal distance between dots was one centimeter (each dot had at least one dot at a distance of 1 one centimeter), and subsets were separated from each other by a fixed distance of four centimeters (i.e., the distance between the two closest dots belonging to two different subgroups was four centimeters). In the “color” grouping-cue condition, the dots were not spatially separated (they all were at a fixed minimal distance of one centimeter, as explained above), but appeared in two, three, or four spatially contiguous subsets, each painted in a different color (black, red, green, or blue, randomly chosen).

The four levels for the grouping-pattern factor were:

1)  The “no-groups” condition: dots were not divided into subgroups. Each array comprised spatially contiguous and equidistant dots. In the “distance” condition, all dots were black on a white background. In the “color” condition, dots were presented in randomly assigned, spatially intermixed colors (see Figure 1).
2)  The “maximally different groups” condition: the array was divided into subgroups which, inasmuch as possible, comprised a different number of dots (4 = 3 + 1; 5 = 3 + 2; 6 = 3 + 2 + 1; 7 = 4 + 3; 8 = 4 + 3 + 1; 9 = 4 + 3 + 2; 10 = 4 + 3 + 2 + 1; 11 = 4 + 3 + 2 + 2; 12 = 4 + 3 + 3 + 2). This condition was designed to induce addition, but not multiplication.
3)  The “minimally different groups with different shape” condition: the array was divided into subgroups with, inasmuch as possible, the same number of dots, yet a distinct spatial arrangement (4 = 2 + 2; 5 = 2 + 2 + 1; 6 = 3 + 3 or 2 + 2 + 2; 7 = 3 + 3 + 1; 8 = 4 + 4 or 2 + 2 + 2 + 2; 9 = 3 + 3 + 3; 10 = 3 + 3 + 3 + 1; 11 = 3 + 3 + 3 + 2; 12 = 4 + 4 + 4 or 3 + 3 + 3 + 3). This condition was designed to induce addition and/or multiplication.
4)  The “minimally different groups with same shape” condition: this was similar to the previous condition, except that the same spatial arrangement was used within each subset (see Figure 1), thus maximally facilitating a multiplication process.

Each array numerosity appeared six times in each condition (with the actual disposition of the dots varying on each trial in order to avoid learning effects). The arrays of 6, 8, and 12 dots, as pointed above, had two different configurations for the third and the fourth condition, since they are divisible in two different configurations having subsets with the same amount of dots (6 = 3 + 3 or 2 + 2 + 2; 8 = 4 + 4 or 2 + 2 + 2 + 2; and 12 = 4 + 4 + 4 or 3 + 3 + 3 + 3); for these arrays, both configurations in third and fourth conditions were presented six times each.

All stimuli were previously generated according to the aforementioned characteristics using a custom program in Python. They were presented in a random order and with a random orientation for each subject.

### Experimental Procedure

On each trial, subjects saw an array that remained on screen. They were explicitly informed that arrays could range from one to 15 items. They were asked to vocally name its numerosity aloud as fast and as accurately as they could. Once they gave their answer, they pressed the spacebar to move to the next trial. If the spacebar was not pressed, the trial automatically ended after 4 s (which was, therefore, the time limit for the vocal response). After each trial, a fixation cross appeared for 1,000 ms at the center of the screen, and then the next array appeared. The duration of the task was ∼50 minutes (three blocks of 15 minutes each, with a 2-min break between them). The six trials per experimental condition were randomly distributed across the three blocks, in order to intermix the presentation of all experimental factors. The subjects performed 50 practice trials in the presence of the researcher before starting the actual experiment, in order to check if they made any sort of mistake (e.g., pronouncing irrelevant words or pressing the spacebar before saying the numerosity, changing their distance from the screen, finger counting, etc.). At the end of the experiment, subjects were asked to freely describe the computation strategies they used in the task, if any.

### Measurement of Vocal Onset

The first author manually detected the vocal onset (together with the accuracy: wrong or right answer) by directly looking at the spectrogram of the recorded vocal response on each trial. In order to avoid any sort of experimental bias, he was not aware of the specific condition of the trial, but only of the target numerosity. This method of measurement of vocal onset, although highly time-consuming, is still considered the gold standard in the literature (Jansen & Watter, 2008; Protopapas, 2007; Roux et al., 2017). Automatized measures gave similar, though less accurate, results.

### Analysis

Median response times (for correct answers) and accuracy were computed for each subject and each cell of the design and entered into either a mixed-model repeated measures omnibus ANOVA with Greenhouse-Geisser sphericity correction, or a linear mixed effect model (see following). Only answers given within 4 s from the stimulus onset were recorded and considered for analysis. Null responses were considered as incorrect answers.

## RESULTS

### Subitizing

Although numerosities 1, 2, and 3 were not part of the main factorial design, we first verified the presence of a classical subitizing effect, that is, virtually identical response times for arrays of one, two, and three dots. The means of median response times were 0.71, 0.70, and 0.73 s, respectively, for arrays of one, two, and three dots, with no significant difference within each numerosity as a function of grouping cue, grouping pattern, or mathematical knowledge (all related *p* values < .01).

### Counting and Groupitizing

**Figure 2.**
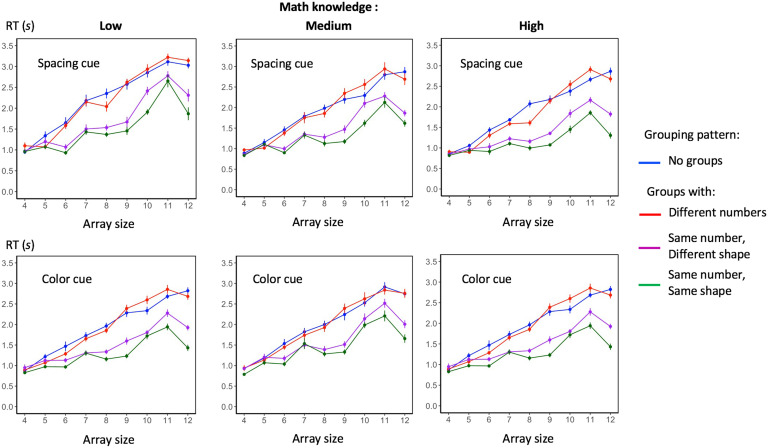
**Mean enumeration times in each condition.** Each graph shows the mean response times for a given group of subjects and a given grouping cue (dots grouped by spacing or by color), as a function of the numerosity of the array (*x*-axis) and the grouping pattern (color legend). Error bars indicate one standard error of the mean.

The main target of our experiment was the existence of a groupitizing effect for larger numerosities in the range 4–12. The corresponding mean RTs appear in Figure 2. As expected, the ANOVA (Table 1) showed a main effect of set size, reflecting the fact that enumeration latencies generally increased with set size. Also, there was a main effect of grouping pattern, and an array size x grouping pattern interaction. To evaluate how grouping pattern affected response latencies, we performed a post hoc Tukey test, which showed that there were significant differences between all four conditions (all *p* < .0001) except for the “no-groups” (mean RT = 2.02 s) versus “maximally different groups” (mean RT = 2.0 s). As predicted, a large acceleration of responses occurred when the array was divided into equal subgroups with the same or maximally similar numerosity (mean RT = 1.58 s), and a significant additional acceleration of naming responses was seen when the same exact shape was used to display each subset (mean RT = 1.39 s). Those results thus indicate a groupitizing effect.

**Table 1.** Mixed model repeated measures omnibus ANOVA

**Effect**	***df***	***F***	***p***	**Partial *η*^2^**
Array size	3.27, 107.92	525.52	<.0001	.94
Grouping cue	1, 33	72.60	<.0001	.69
Grouping pattern	2.01, 66.42	286.77	<.0001	.90
Array size*Grouping cue	3.32, 109.61	2.94	.03	.08
Array size*Grouping pattern	5.09, 167.85	27.15	<.0001	.45
Grouping cue*Grouping pattern	2.39, 78.79	5.87	.003	.15
Array size*Grouping cue*Grouping pattern	4.65, 153.37	3.77	.70	.06
Math knowledge	2, 33	8.43	.001	.34
Math knowledge*Array size	6.54, 107.92	2.34	.03	.12
Math knowledge*Grouping cue	2, 33	0.69	.51	.04
Math knowledge*Grouping pattern	4.03, 66.42	0.86	.49	.05
Math knowledge*Array size*Grouping cue	6.64, 109.61	1.95	.07	.11
Math knowledge*Array size*Grouping pattern	10.17, 167.85	1.88	.05	.10
Math knowledge*Grouping cue*Grouping pattern	4.78, 78.79	0.56	.72	.03
Math knowledge*Array size*Grouping pattern *Grouping cue	9.29, 153.37	1.10	.37	.06

### Effect of Grouping Cue

We then looked at the effects that involved grouping cue (color or spatial distance). In the main ANOVA, a main effect of grouping cue and a grouping cue *x* grouping pattern interaction were found, indicating that participants were slightly faster with spatial cues than with color cues overall, Welch *t* test, *t*(2589.6) = 2.98, *p* < .01; mean RTs, respectively, 1.71 s and 1.79 s, and had slightly greater savings from spatial cues when these afforded equal groups, Welch *t* test, *t*(1292.4) = −4.02, *p* < .001; mean RT for arrays with spatial cues: 1.43 s; with color cues: 1.55 s. Nevertheless, both color and spatial cues made groupitizing possible: as shown in Figure 2, very similar profiles of responses were found for both. We separately submitted the “color” condition and the “spacing” condition to mixed models repeated measures ANOVAs and we found the same main effects and interaction effects discussed above for both grouping cues.

### Effect of Specific Numerosities

Since there was an array size *x* grouping pattern interaction, we next examined how response times varied with numerosity in each condition. For the no-groups and the different-numbers conditions, response times increased roughly linearly with numerosity, a classical phenomenon that reflects the serial process of counting (Dehaene, 1992; Moyer & Landauer, 1967) for sets comprising more than three items (Dehaene & Cohen, 1994). However, in the condition of grouping by equal or maximally similar numbers, the effect of array size ceased to be monotonic and roughly linear (see Figure 2). Instead, there was an acceleration of responses that was most pronounced for nonprime numbers that could be subdivided in equal numbers. Conversely, the prime numbers 5, 7, and 11 were slower than their neighbors, leading to reversals in monotonicity. Thus, sets of five items were enumerated more slowly than sets of six items, Welch unequal variances *t* test on response times, *t*(285.92) = 2.18, *p* = .03; mean RTs, respectively, 1.11 s and 1.04 s, sets of seven items more slowly than sets of eight items, *t*(285.5) = 2.44, *p* = 0.015; 1.42 s versus 1.32 s, and sets of 11 items more slowly enumerated than sets of 12 items, *t*(285.77) = 9.14, *p* < .0001; 2.36 s versus 1.85 s. This pattern, which was present in all six groups *x* cue conditions (Figure 2), indicates that numerosities that could be resolved by multiplication alone (e.g., 8 = 4 groups of 2) were faster than numerosities that required a combination of addition and multiplication (e.g., 7 = 3 groups of 2 plus a group of 1).

As a quantitative test of this idea, we performed a linear mixed effects analysis, where the dependent variable was the mean response time to each numerosity in the range 4–12 in the “equal groups” conditions, and the fixed effects were array size (as a proxy for problem size, which is a good predictor of multiplication difficulty), and the type of arithmetic operation postulated under our hypothesis: 0 for a simple multiplication (such as for 4 = 2 × 2), 1 for a multiplication and an addition of 1 (such as for 5 = 2 × 2 + 1), 2 for a multiplication and an addition of 2 (only for 11 = 3 × 3 + 2). The subjects were included as random effects. For the “equal groups” condition, both array size, slope = 139 ± 4 ms/item [±standard error], *t*(286) = 35.25, *p* <10^−16^, and operation type, slope = 289 ± 14 ms per additional added item, *t*(286) = 19.41, *p* <10^−16^, were highly significant (conditional *r*^2^ = 89.5% of variance explained), thus confirming that the maximal savings were observed for nonprime numbers that could be subdivided into equal groups, and that the need to add 1 or 2 imposed an additional toll of almost 300 ms/item for numerosities 5, 7, 10, and 11. When we performed a similar regression on the mean RT from the “unequal or no groups” conditions, array size had a dominant effect, slope = 247 ± 6 ms/item, *t*(286) = 40.79, *p* <10^−16^, while operation type had a much less pronounced, though still significant effect, slope = 68 ± 22 ms/item, *t*(286) = 2.97, *p* = .0032. Thus, groupitizing effects were perhaps not totally absent even when the arrays were not systematically arranged as equal groups, but became very prominent in the “equal groups” conditions.

### Order Effect

**Figure 3.**
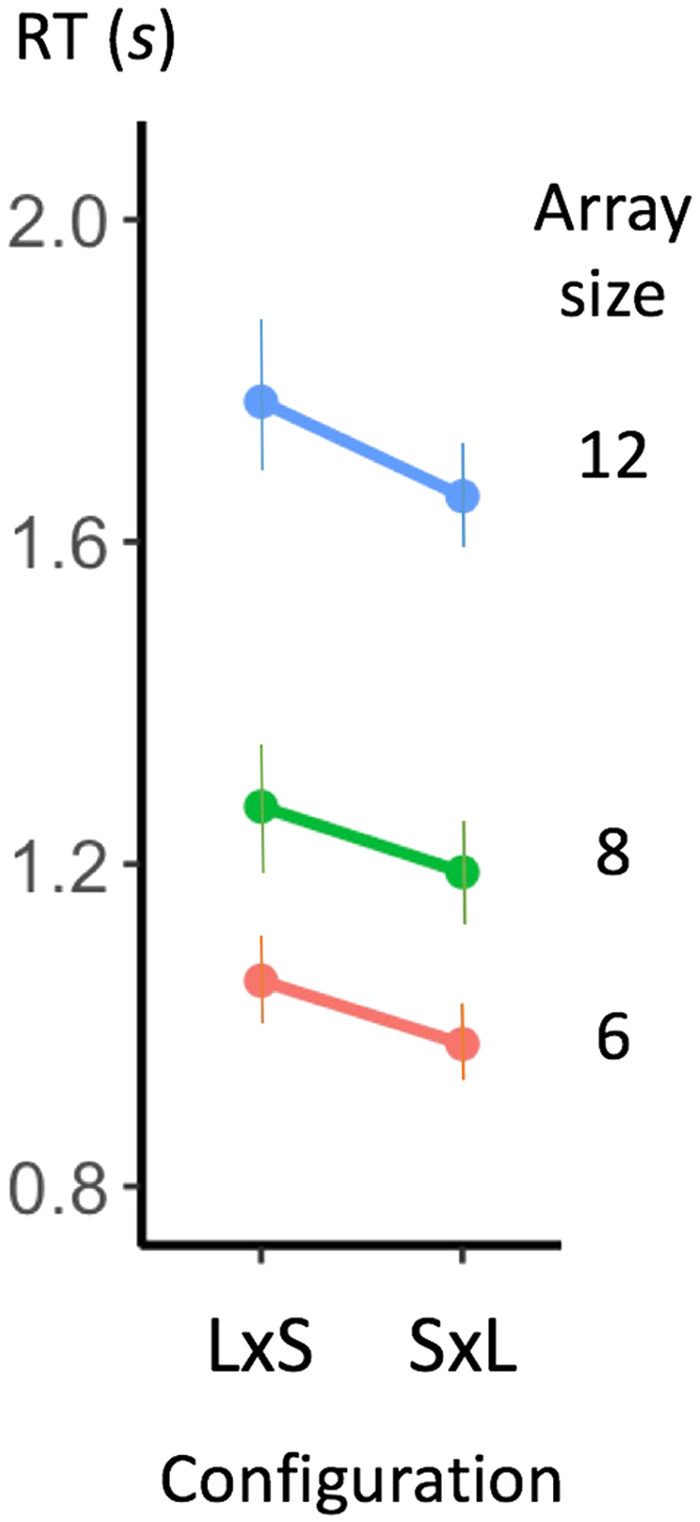
**Influence of grouping order on enumeration speed.** For numerosities 6, 8, and 12, which could be grouped in two different ways, response times were slower in the configuration LxS, with the largest number of groups and the smallest number of dots per group (e.g., 6 = 3 groups of 2 dots) than in the configuration SxL, with the smallest number of groups, and the largest number of dots per group (e.g., 6 = 2 groups of 3 dots). Error bars indicate one standard error of the mean.

In the conditions with the same number in each group, arrays of six, eight, and twelve dots offered another test of the multiplicative model. Such arrays were presented to the subjects in two possible configurations, reflecting the commutativity of multiplication: the number of groups could be either the smaller number (for example, eight dots divided into two groups of four dots) or the larger number (for example, eight dots divided into four groups of two dots). A classical finding in symbolic arithmetic is that there is an order effect in mental multiplication (Aiken & Williams, 1973; Dehaene, 1992; Zimmerman et al., 2016): it is easier to compute a multiplication such as 8×3 as three groups of eight (3 times 8 in English) than as eight groups of three (8 times 3 in English). If there was a covert multiplication during enumeration, then this effect should also appear in our data. Indeed, we found that the first type of configuration, with a smaller number of groups, led to significantly faster responses; in other words, response times increased with the number of groups more than with the number of items in a group (see Figure 3). Welch unequal variances *t* test were conducted for each array size, showing a significant difference between the two configurations for all array sizes, array of 6: *t*(279.4) = 2.88, *p* < .01; array of 8: *t*(272.64) = 2.88, *p* < .01; array of 12: *t*(274.15) = 2.10, *p* < .001.

### Influence of Math Knowledge

The ANOVA on RTs also revealed a significant effect of mathematical knowledge and its interactions with array size and with grouping pattern (see Table 1). We thus submitted the results to a post hoc Tukey test, which revealed no significant difference in response times between the two groups of students from the most selective university (high and medium math knowledge; *t* ratio = 1.13, *p* = .5) but a significant difference between the group of low-math students (mean RT = 1.932 s) and both the medium (mean RT = 1.705 s; *t* ratio = 2.85, *p* = .019) and high one (mean RT = 1.615 s; *t* ratio = 3.98, *p* = .001). An interaction of mathematical knowledge and array size, as well as a triple interaction of math knowledge, array size and grouping pattern were also found. To shed light on those effects, we conducted four separate ANOVAs on response times, one for each grouping pattern, with array size as within-subjects factor. Mathematical knowledge, as apparent from Figure 2, had a significant main effect on response times for all grouping patterns (all related *p* < .01): students in the low-level group were overall slower than the other ones. Crucially, for the conditions with no groups and with different groups, mathematical knowledge did not interact with the array size effect, respectively: *F*(6.16, 101.71) = 1.14, *p* = .35; *F*(4.43, 73.05) = 1.06, *p* = .39. As indicated by the parallel blue and red curves in all panels of Figure 2, all participants were equally skilled in counting. However, we found significant interactions of math knowledge and array size in both the same groups/different shape and the same groups/same shape conditions, respectively: *F*(8.05, 132.78) = 4.47, *p* < .0001; *F*(8.01, 132.20) = 6.28, *p* < .0001. This observation fits with the idea that, for arrays divided into equal groups, participants were computing a mental multiplication, and that this capacity was modulated by their level of math knowledge.

### Testing of an Alternative to the Multiplication Hypothesis

One could argue that the results are compatible with an alternative skip-counting strategy: instead of a multiplication (e.g., 6 = 3 × 2), subjects would always count, but the grouped format would afford a faster process of counting by multiples of two or three (e.g., 6 = 2, 4, **6**). This model, however, can be rejected. The skip-counting strategy predicts that arrays with (a) equal subgroups of two items (2 × 2, 3 × 2 and 4 × 2) or with (b) equal subgroups of three items (2 × 3, 3 × 3, 4 × 3) should exhibit a linear increase in RTs, comparable to the one observed for arrays in the no-groups condition (c), for which subjects are expected to count by one. A linear regression on data from (a) did show a significant slope of 130 ± 12 ms/item [±standard error], *t*(214) = 10.49, *p* <10^−16^, as did a similar regression on data from (b) slope 116 ± 9 ms/item [±standard error], *t*(214) = 12.64, *p* <10^−16^, but those slopes were consistently smaller than that observed in the data from (c) slope of 256 ± 5 ms/item [±standard error], *t*(646) = 46.16, *p* <10^−16^. If anything, this should be the opposite: counting by twos or threes should be slower than counting by ones. Thus, the increment in RT observed for multiples of two and three is too small to be compatible with a skip-counting strategy, and is more likely to arise from the known effect of problem size on multiplication times.

An even simpler argument against the skip-counting strategy arises from a comparison of RTs in the same-groups versus no-groups conditions, between arrays that, according to the skip-counting hypothesis, should be based on the same number of counting steps. For instance, 8 = 4 × 2 in the same group condition (i.e., 8 = 4, 6, **8** according to skip counting; we start from four since, in all conditions, the RT to four items is essentially the same, in agreement with classical observations on subitizing) should reveal the same RT for six items in the no-groups condition (i.e., 6 = 4, 5, **6** according to simple counting). This is a conservative hypothesis in two ways. First, it assumes that counting by ones or by twos takes the same amount of time; if counting by twos takes longer, the RT in the same groups condition should be even slower. Second, it assumes that subjects count by ones in the no-group condition; otherwise, again, the RT in the no-group should be even faster (as the proper comparison should be with the RT to 8 in the no-group conditions, assuming that subjects arrive at it by computing 8 = 4, 6, **8**; but then there should really be no same-group advantage).

In summary, the skip-counting hypothesis predicts that RT_no groups_(6) ≤ *RT*_same groups_ (4 groups of 2). But the opposite is true, as it is apparent in Figure 2 (the blue point for 6 compared with the green point for 8), and the difference is significant in the wrong direction, mean_6_ = 1.56 s, mean_8_ = 1.38 s, *t*(142) = 2.63, *p* < .01. Similarly, we found that RT_no groups_ (5) > RT_same groups_ (6 = 3× 2), mean_5_ = 1.24 s, mean_6_ = 1.03 s, *t*(138.76) = 4.21, *p* < .0001; RT_no groups_ (6) > RT_same groups_ (9 = 3 × 3), mean_6_= 1.56 s, mean_9_= 1.31 s, *t*(132.65) = 4.14, *p* < .0001; and RT_no groups_ (7) > RT_same groups_ (12 = 4×3), mean_7_= 1.89 s, mean_12_= 1.63 s, *t*(140) = 3.88, *p* < .001. In other words, all these relationships are in the opposite direction of the predictions of the skip-counting hypothesis. To put it bluntly, subjects are simply too fast in the same-group condition for it to be compatible with a slow serial counting–based strategy.

### Error Patterns

**Figure 4.**
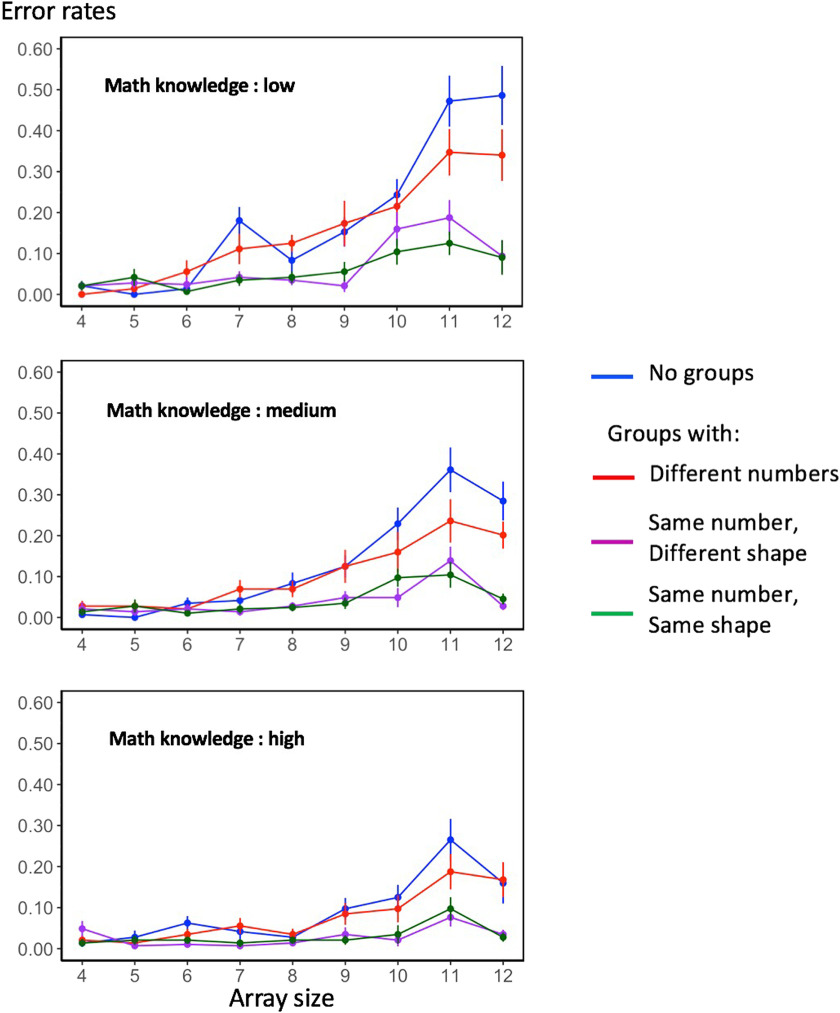
**Mean error rates as a function of the numerosity of the array (*x*-axis), the grouping pattern (color legend), and mathematical knowledge (three groups of subjects).** Error bars indicate one standard error of the mean.

Overall accuracy was very high (>90%), which was expected given that subjects had up to 4 s to respond. Nevertheless, we submitted the mean error rates per condition to the same ANOVA conducted on response times, and found the same main effects except for the absence of a grouping cue effect, array size: *F*(2.33, 76.91) = 45.94, *p* < .0001; grouping pattern: *F*(1.89, 62.23) = 33.20, *p* < .0001; mathematical knowledge: *F*(2, 33) = 3.91, *p* = .03; grouping cue: *F*(1, 33) = 1.67, *p* = .02. There was an interaction of mathematical knowledge *x* array size, *F*(4.66, 76.91) = 2.99, *p* = .02, and no interaction between mathematical knowledge and the grouping pattern, *F*(3.77, 62.23) = 1.20, *p* = .32. Figure 4 shows, as a summary, the mean error rates per condition (independently on the grouping cue), separately for the three levels of the mathematical knowledge factor. As we can easily see, the higher the mathematical knowledge, the smaller the number of errors, especially for large arrays.

Once again, we conducted a post hoc Tukey test to evaluate how the grouping pattern affected error rates: we found significant differences between all conditions (all *p* < .0001) except for the “no-groups” versus “maximally different groups” (*t* ratio = − 2.02, *p* = .1867) and for the two conditions with equal (or maximally similar) numbers of dots per subgroup (*t* ratio = 0.18, *p* = .9979). In the latter conditions, errors dropped almost to zero, except for the numbers above ∼8 (Figure 4). We next analyzed the nature of those remaining errors.

### Distribution of Errors

**Figure 5.**
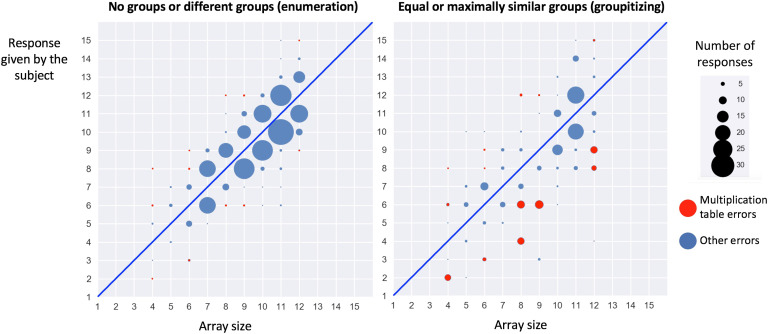
**Distribution of enumeration errors as a function of the grouping pattern (left graph: no groups and groups with different numbers of dots; right graph: groups with the same or maximally similar numbers of dots).** Note how the distribution of errors changes with grouping: not only do errors massively decrease, but the distribution ceases to be dominated by nearest-neighbor errors (target ± 1) and many more multiplication table errors occur.

The hypothesis that subjects used a multiplicative shortcut in the “equal groups” conditions can be tested by examining the distribution of errors. Figure 5 shows how errors were distributed, separately for arrays with no grouping or divided into different groups (left graph) and with equal or maximally similar groups (right graph). In the former case, erroneous responses mostly corresponded to numbers close to the correct array size, often within a distance of ±1, suggesting that they correspond to counting errors. However, in the “equal groups” conditions (right graph), the distribution differed: errors were often more distant from the target and, furthermore, often corresponded to another number within the same row or column of the multiplication table (e.g., for an array of 9 presented as three groups of three, the errors were often 6 or 12). Focusing solely on the array sizes 4, 6, 8, 9, and 12, a simple count confirmed that such “table errors” were significantly more frequent in the “equal groups” conditions (81/136 = 59.56%) than in the other conditions (20/188 = 10.63%; *χ*^2^ = 85.758, *df* = 1, *p* < .0001), whereas the converse was true for close errors (i.e., correct numerosity ± 1): they were significantly less frequent in the “equal groups” conditions (43/136 = 31.62%) than in the other conditions (145/188 = 77.13%; *χ*^2^ = 65.25, *df* = 1, *p* < .0001). This finding thus supports the hypothesis that a multiplication process underlies the savings in enumeration time that characterize the groupitizing phenomenon.

## DISCUSSION

Given the results presented in the previous section, it is now possible to answer the questions formulated at the beginning of the article.

1) Do repeated groupings with the same number of items facilitate groupitizing? The results indicate that the presentation of repeated patterns of items facilitate groupitizing. Arrays divided into equal subsets were systematically enumerated faster and more accurately than arrays divided into nonequal subsets. This effect was maximal when the subgroups shared not only the same numerosity, but also the same shape, and thus promoted a multiplication process.

More surprisingly, the arrays divided into subgroups with unequal numbers of dots (e.g., nine dots divided into three subgroups of four, three, and two dots) were not enumerated faster or more accurately than arrays with no grouping cue. According to previous literature (Mandler & Shebo, 1982; Starkey & McCandliss, 2014; Wender & Rothkegel, 2000; Wolters et al., 1987), we should have expected an advantage for sets organized in subgroups, regardless of their number of items. One interpretation of this negative result is that our adult participants were, in fact, already making use of counting-by-groups even in the no-group condition. Indeed, most of the subjects (29 out of 36), when asked about the perceived strategies used in the task, explicitly referred to the autonomous formation of subgroups in the “no-groups” condition: they actually tried to form some small and mostly subitizable groups of dots in order to accelerate the counting process. Therefore, an internally driven grouping mechanism might have facilitated subjects’ responses, making their response times in the “no-groups” condition (blue line in Figure 2) not significantly different from the response times in the “maximally different groups” condition (red line in Figure 2). Our results indicate that, in educated adults, seeing an array decomposed in subcomponents does not necessarily represent a benefit, both in terms of response times and accuracy, for numerosity visual detection, because an internally driven grouping mechanism may be just as fast and efficient as an externally driven one. This hypothesis is in agreement with many studies showing a link between arithmetic abilities and numerosity estimation proficiency (DeWind & Brannon, 2012; Halberda et al., 2008; Halberda et al., 2012; Lyons & Beilock, 2011) since it shows how even a very simple enumeration task might provide a surreptitious assay of mental arithmetic through the mediation of an internally driven grouping process. However, more research will be needed to confirm our hypothesis of an internally driven grouping mechanism being at play, and its role in numerosity estimation.

2) Can color act as a groupitizing cue? According to our results, color is just as good as spatial distance in supporting groupitizing. Arrays with contiguous dots grouped by color were enumerated faster than the same arrays where the colors were randomly dispersed. Furthermore, over all conditions of the experiment, the results were remarkably parallel whether color or space was used as a grouping cue (Figure 2). Thus, groupitizing is a robust phenomenon, regardless of whether color or spacing is used as the grouping cue. This result is coherent with the scientific literature on the role of Gestalt factors on perception and attention (Brunswik & Kamiya, 1953; Wagemans et al., 2012; Wolfe et al., 1989) and with the human capacity to immediately access the numerosity of items that are selected on the basis of their color (Halberda et al., 2006). Note that Halberda et al. (2006) found that most subjects could not estimate more than three such groups in parallel, whereas our displays contained up to four groups; however, our results do not necessarily contradict theirs, since contrary to Halberda et al.’s paradigm, our task did not enforce parallel processing of the groups (and the RTs in Figures 2 and 3 do suggest a significant degree of serial processing).

3) Which cognitive computations underlie groupitizing? Our results clarify which cognitive computations are used in groupitizing. They suggest that grouping is particularly useful when the subgroups allow for a multiplication or a combination of multiplication and addition. When at least one multiplication could be used (in the conditions with “minimally different groups”), the subjects enumerated the arrays faster relative to the condition with “maximally different groups,” which did not allow for any multiplication. Further support for this conclusion comes from the observation that smaller sets were sometimes enumerated more slowly than larger sets, whenever the latter could be grouped in the most regular manner. Thus, sets of five items were enumerated more slowly than sets of six items, seven items more slowly than eight items, and 11 items more slowly than 12 items, in the conditions where the subgroups shared, inasmuch as possible, the same numerosity. The explanation is simple: numbers 5, 7, and 11 are prime and therefore cannot be subdivided into equal subgroups, hence, they could not elicit a single multiplication but a combination of multiplication and addition (e.g., 7 = 2 × 3 + 1); on the contrary, sets of six, eight, and twelve items could be enumerated through a shortcut based on a single multiplication (e.g., 8 = 2 × 4). In hindsight, precursors of this result can be found in the literature. Starkey and McCandliss (2014) only collected data for arrays of five, six, or seven elements, each made of three subitizable subgroups, and their figures show that participants were faster at enumerating arrays of six elements compared to arrays of five and seven dots (which are prime). Likewise, Mandler and Shebo’s (1982) results, replicated by Wender and Rothkegel (2000), show that arrays of six, eight, and nine dots (organized in canonical patterns) were enumerated considerably better and faster than arrays of seven or 10 dots. For the latter, the subjects were forced to compute, respectively, 2×3 + 1 and 2×4 + 2, whereas arrays of six, eight, and nine were arranged as 2×3, 2×4, and 3×3 matrices of dots—again supporting a simple multiplication process.

Further support for the mental multiplication hypothesis comes from two additional observations. First, on groupitizing trials, subjects often made enumeration errors that fell within the correct row or column of the multiplication table (e.g., answering six instead of nine), as if a mental multiplication was autonomously elicited (Zbrodoff & Logan, 1986). Second, with arrays of six, eight, and twelve dots, the smaller the number of subgroups, the faster the response times. For instance, subjects were faster with two groups of three dots compared to three groups of two dots. The crucial point is that the same errors and asymmetries are observed during mental calculation with Arabic digits (Aiken & Williams, 1973; Campbell & Graham, 1985; Zimmerman et al., 2016).

We also investigated an alternative to the mental multiplication hypothesis: skip-counting, whereby subjects would count by twos or by threes in the same-groups condition. Several observations argue against the skip-counting model. First, the increment in RTs with each additional group of items is twice as big for arrays that cannot be divided in groups compared to those divided in groups of two or three items. In fact, for the skip-counting hypothesis to hold, we should have found comparable RTs for arrays promoting counting by ones, by twos and by threes or, if anything, faster counting by ones—the opposite of the results. Second, we observed that, for the same number of counting steps, arrays divided in equal groups were enumerated faster (for instance, the RTs for eight items in the same-groups conditions were significantly faster than the RTs for six items in the no-groups condition). Third, the interaction of mathematical knowledge with array size was significant only for the arrays divided in equal groups, an observation that would make no sense if subjects were using counting in all cases, but does make sense if they were using multiplication only for the latter, and differed in their multiplication skills. Finally, the error pattern was characterized by slips in the multiplication table, as expected from a multiplication process.

Note that none of these arguments against skip counting are absolutely definitive: it could be that, even in the no-groups condition, subjects also count by twos or threes, only with a steeper slope due to the greater difficulty of forming the groups internally instead of seeing them on screen. It could be that only the external grouping, but not the internal grouping, is faster in mathematically more advanced students (though it is not clear why this would be the case). Finally, errors in skip counting, such as stopping the count too early or too late, could account for the error pattern (though it is not clear why such errors would not occur in the no-groups or different-groups condition). All in all, however, and pending further research, we find that the multiplication hypothesis provides a more parsimonious account of all the data. We conclude that a visual grouping format that facilitates the conceptualization of a number as being composed of a small number of equal subgroups facilitates the enumeration process, probably through the mediation of a multiplication process.

4) Does groupitizing vary with mathematical knowledge? We found a significant effect of mathematical knowledge, both in response times and in accuracy. A significant interaction of mathematical knowledge with array size and a triple interaction of math knowledge *x* array size *x* grouping pattern, were also observed, and turned out to reflect a specific reduction of the array size effect in the conditions that promoted groupitizing. Previous studies have shown that mathematical training can enhance the precision of nonsymbolic number estimation (Piazza et al., 2013) and, vice versa, have suggested that training the latter can improve math proficiency (Park & Brannon, 2013). Our findings suggest that mathematical training also enhances the precision of enumeration, with a specific improvement for configurations that promote a multiplication strategy. Nevertheless, groupitizing effects were present and showed very similar qualitative profiles in all three groups, suggesting that arithmetic shortcuts can be used as long as basic arithmetic has been acquired, independently of the knowledge of higher mathematical concepts (Starkey & McCandliss, 2014). It would be interesting to replicate the present experiment in a younger sample, where there could be greater variability in elementary mental arithmetic abilities.

## CONCLUSION

Overall, our findings confirm that arithmetic knowledge such as 3 × 3 = 9 can be probed by an elementary numerosity naming task, in agreement with the literature showing that the performance in elementary numerosity detection or comparison tasks correlates with arithmetic skills (Piazza et al., 2010; Starkey & McCandliss, 2014). Furthermore, our findings support the hypothesis that different cognitive computations are used depending on the grouping pattern, thus pointing to the precise conditions under which grouping may or may not be beneficial in numerosity detection tasks: when the subgroups are equal, mental multiplication allows subjects to be faster and more accurate at determining the numerosity of the array but, in case of unequal subsets, this groupitizing advantage is reduced.

## ACKNOWLEDGMENTS

We are thankful to the Département d’Etudes Cognitives of Ecole Normale Supérieure de Paris for hosting our experimental sessions. We are grateful to Michele Orrù (Ecole Normale Supérieure de Paris) for his help with stimuli creation and to Marie Lubineau (ESPCI Paris) for her help with data collection.

## FUNDING INFORMATION

SD, INSERM, CEA, Collège de France, and the Bettencourt-Schueller foundation. LC, Ministère de l’Enseignement supérieur, de la Recherche et de l’Innovation (France) and the Ecole Doctorale FIRE - Programme Bettencourt.

## AUTHOR CONTRIBUTIONS

LC: Conceptualization: Equal; Data curation: Lead; Formal analysis: Equal; Methodology: Equal; Visualization: Equal; Writing: Equal. SD: Conceptualization: Equal; Formal analysis: Equal; Methodology: Equal; Visualization: Equal; Writing: Equal.
